# Supplementary material for: Community Structures and Antifungal Activity of Root-Associated Endophytic Actinobacteria in Healthy and Diseased Cucumber Plants and Streptomyces sp. HAAG3-15 as a Promising Biocontrol Agent
Source: Microorganisms. 2020 Feb 10;8(2):236. doi: 10.3390/microorganisms8020236 (PMC7074843; doi:10.3390/microorganisms8020236)
Supplement: Supplementary file 1 [file microorganisms-08-00236-s001.pdf]

Community Structures and Antifungal Activity of Root-Associated Endophytic Actinobacteria in Healthy and Diseased Cucumber Plants and *Streptomyces* sp. HAAG3-15 as a Promising Biocontrol Agent

Peng Cao [1,†], Chenxu Li [1,†], Han Wang [1], Zhiyin Yu [1], Xi Xu [1], Xiangjing Wang [1], Junwei Zhao [1,*] and Wensheng Xiang [1,2,*]

[1] Key Laboratory of Agricultural Microbiology of Heilongjiang Province, Northeast Agricultural University, No. 600 Changjiang Road, Xiangfang District, Harbin 150030, China; cp511@126.com (P.C.); licx95@126.com (C.L.); wanghan507555536@gmail.com (H.W.); yuzhiyin123@foxmail.com (Z.Y.); xuxi1758899581@163.com (X.X.); wangneau2013@163.com (X.W.)

[2] State Key Laboratory for Biology of Plant Diseases and Insect Pests, Institute of Plant Protection, Chinese Academy of Agricultural Sciences, Beijing100193, China

* Correspondence: guyan2080@126.com (J.Z.); xiangwensheng@neau.edu.cn (W.X.)

† These authors contributed equally to this work.

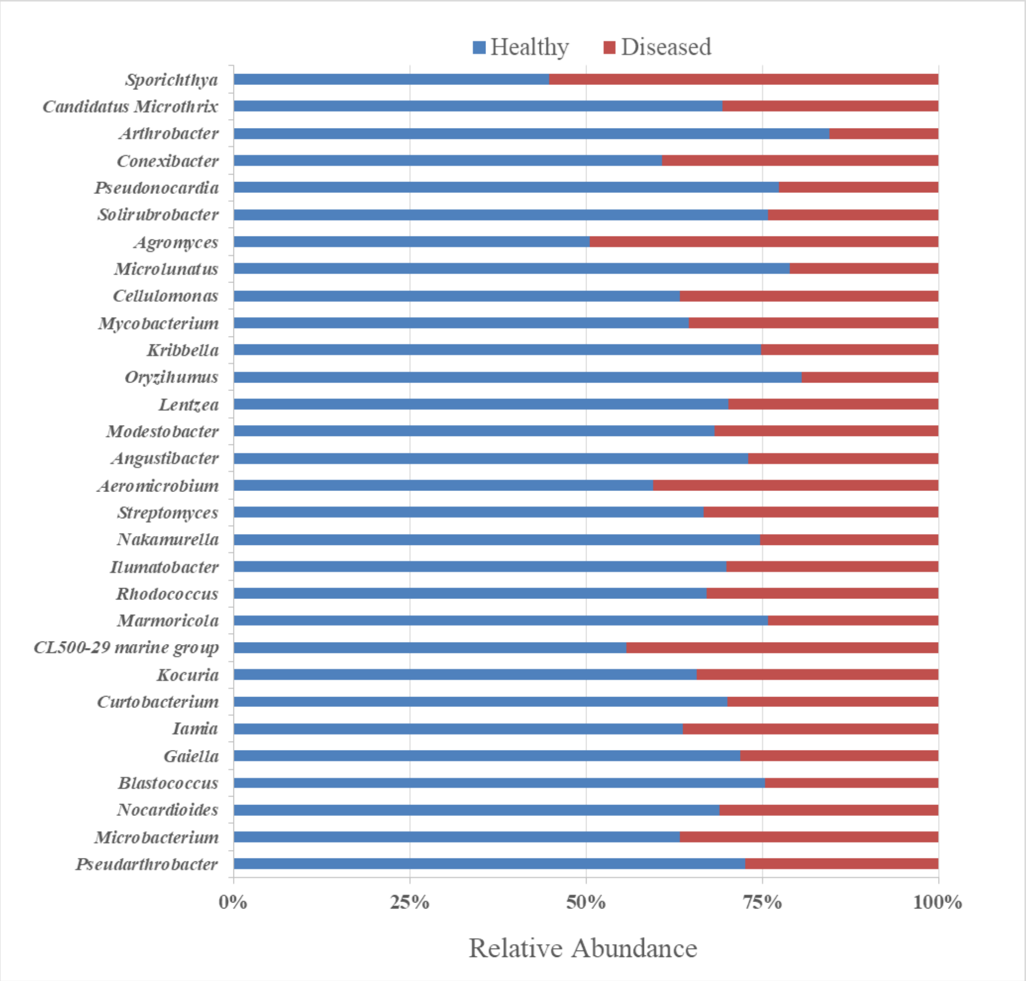

Figure S1. Analysis of culture-independent endophytic actinobacteria communities at genus level top 30 in the cucumber roots.

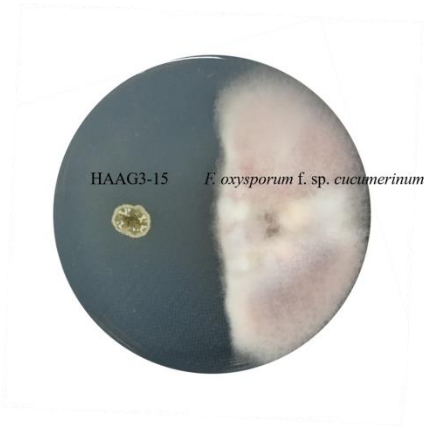

Figure S2. Strain HAAG3-15 showed antagonism to *F. oxysporum* f. sp. *cucumerinum*.

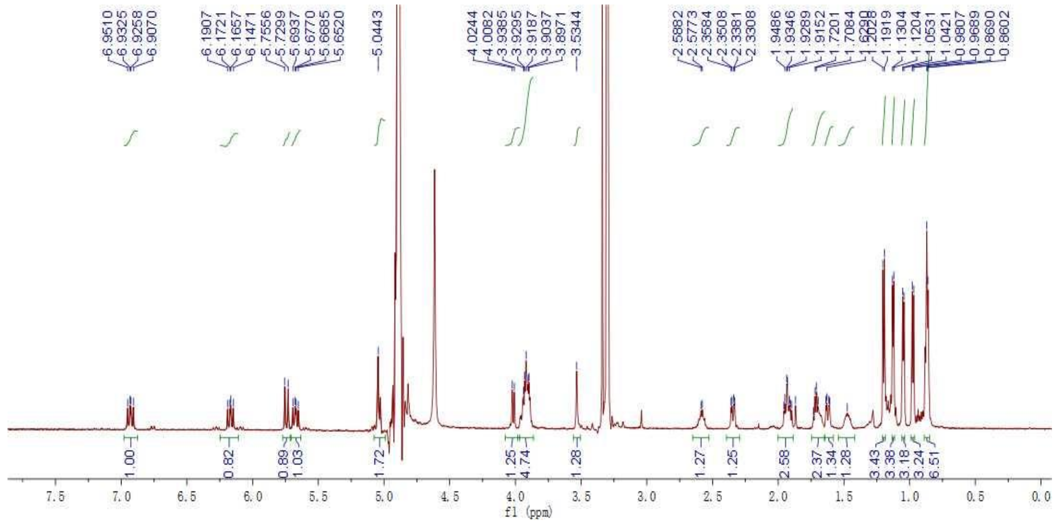

Figure S3. ^{1}H NMR (600 MHz) spectrum of compound 1 in MeOD.

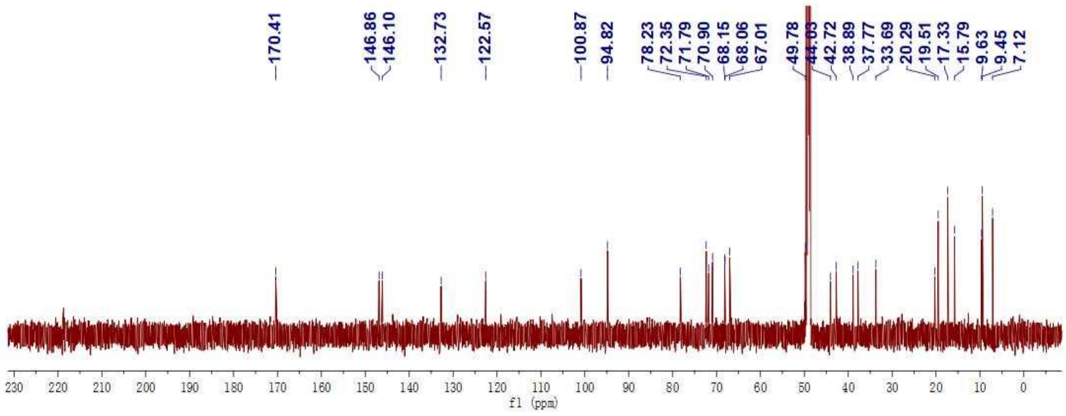

Figure S4. ^{13}C NMR (150 MHz) spectrum of compound 1 in MeOD.

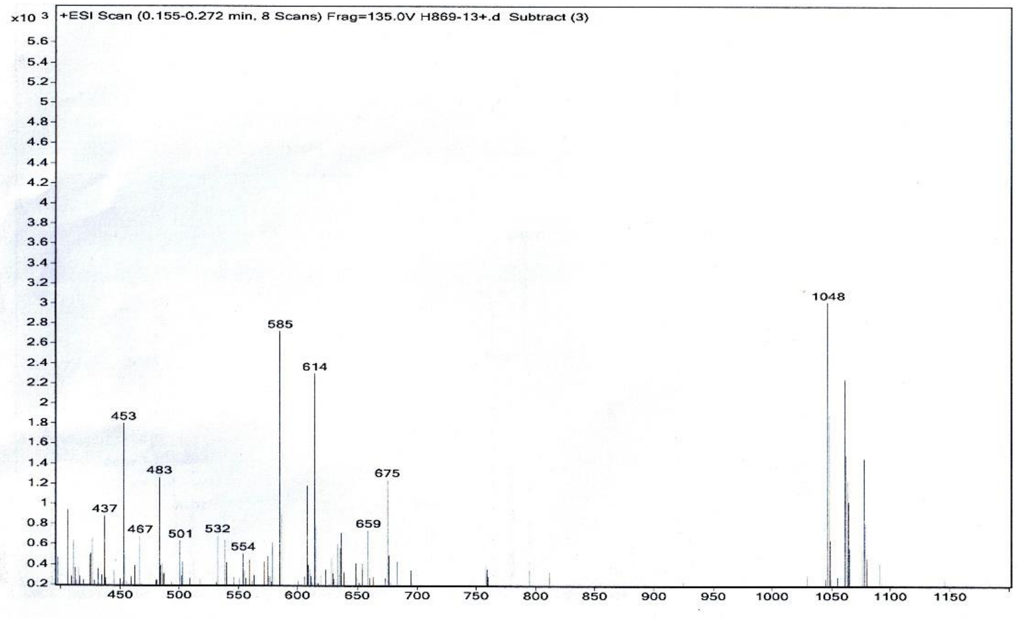

Figure S5. ESI-MS spectrum of compound 1.
